# Anionic Calixarene-Capped Silver Nanoparticles Show Species-Dependent Binding to Serum Albumins

**DOI:** 10.3390/molecules18055993

**Published:** 2013-05-21

**Authors:** Yannick Tauran, Arnaud Brioude, Beomjoon Kim, Florent Perret, Anthony W. Coleman

**Affiliations:** 1 LMI, CNRS UMR 5615, Université Lyon 1, Villeurbanne F69622, France; 2 Institute of Industrial Science, the University of Tokyo, 4-6-1 Komaba, Meguro-ku, Tokyo 153-0041, Japan; 3 ICBMS, UMR 5246, Université Lyon 1, Villeurbanne F69622, France

**Keywords:** calixarenes, molecular recognition, serum albumins, species dependence

## Abstract

The anionic calixarenes *para*-sulphonatocalix[4]arene and 1,3-di-O-phosphonatocalix[4]arene, have been used to cap silver nanoparticles. The binding of these functional particles with regard to various serum albumins (bovine serum albumin, human serum albumin, porcine serum albumin and sheep serum albumin) has been studied by variable temperature fluorescence spectroscopy. The quenching of the fluorescence of the proteins was shown to vary as a function of the anionic calixarene capping molecule and also as a function of the origin of the serum albumin. It is thus possible to discriminate between the different species.

## 1. Introduction

In late 2012 and early 2013, a number of public health scandals erupted in Asia and Europe [1]. The first of these concerned the Chinese subsidiary of a multinational food company; here a plethora of disparate Active Pharmaceutical Ingredients (APIs) were found to be contaminants in chicken meat [2]. They included caffeine, amantadine, an anti-Parkinson AP, antibiotics including the banned fluoroquinolines and intriguingly, Prozac. This represents a true public health safety risk and will be treated in a separate publication. In Europe there was recently a media explosion concerning the presence of horsemeat in a wide range of frozen and prepared beef products [3]. However more careful analysis of the readily available data shows that is a minor problem as there is no true health risk, although the UK there is undoubtedly disgust about the consumption of horse, a widely loved animal. In reality this crisis concerns a fraud of deliberate mislabeling of horsemeat as beef, however it was widely trumpeted by the media and government agencies [4]. Yet there are two more problems associated with contaminated beef products. Firstly, the presence of the banned veterinary API phenylbutazone, as despite government assurances that there the level was not dangerous, the permitted level of this API in the human food chain is zero [5]. The second contamination does not represent a true health risk but is far more serious, as compared to horsemeat, from an ethical point of view as it concerns the contamination by pork of beef products, including in at least one case, a product labeled Halal [6]. Interestingly the Irish Food Safety Agency published an analysis of the presence of pork and horsemeat in frozen beef products. The results are striking, of the 27 products tested only three were not contaminated with meat from other species; only 10 samples showed the presence of horsemeat, however 23 products showed the presence of pork. In view of the above, methods to check for the presence of pork in food products would seem to be useful [7].

Aside from DNA testing, detection of serum albumins would appear to be an interesting route for the investigation of contaminants in food stuffs [8]. The serum albumins represent the most common proteins in animal physiological fluids with up to 40 g per liter being present. This class of proteins is capable of transporting a wide variety of molecules and ions, including copper, various carboxylic acids and a large range of APIs [9]. Recently a crystallographic study on a number of serum albumins has demonstrated that despite a high degree of homology, the binding pockets in these proteins differ both in nature and also in their capacity to bind substrate molecules [10].

The anionic calixarenes have been investigated over a number of years for their capacity to complex biomolecules, including a wide range of proteins [11]. Examples of such complexation include proteins of the blood coagulation cascade [12], ATP binding domains of ABC transporters [13], calcium dependent ion channels [14], histones [15], the protease resistant protein, prion protein [16], other proteins associated with neurodegenerative diseases [17] and especially the serum albumins [18]. Previous work has shown that the *para*-sulphonatocalix[n]arenes interact with several binding sites of the serum albumins but also that such binding depends on the nature of the source of the serum albumins [19]. In this last case the studies used Electrospray Mass Spectrometry under partially denaturing conditions, in view of this we have turned to the use of calixarene-capped silver nanoparticles to generate systems with internal probes for the complexation. Previous work by Xiong on amino acid binding [20] and by ourselves on nucleic acid [21] or API [22] binding has demonstrated the interest of such an approach.

In the current work we treat the dependent temperature binding of bovine serum albumin (BSA), human serum albumin (HSA), porcine serum albumin (PSA) and sheep serum albumin (SSA) with silver nanoparticles capped by *para*-sulphonatocalix[4]arene (**1**_Ag_NP) and 1,3-di-O-phosphonato-calix[4]arene (**2**_Ag_NP) by spectroscopic methods. The results show that the use of only two types of capped nanoparticles allows the discrimination of serum albumins from different mammalian species.

## 2. Results and Discussion

**Scheme 1:**
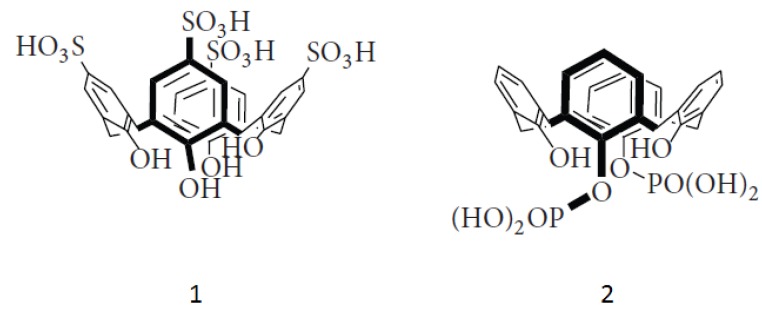
Structure of the anioinic calix[4]arenes studied. Compound **1** is *para*-sulphonato-calix[4]arene and **2** is 1,3-di-O-phosphonato-calix[4]arene.

The molecular structures of *para*-sulphonatocalix[4]arene, (**1**) and 1,3-di-O-phosphonato-calix[4]arene (**2**) are shown below in Scheme 1.

*Para*-sulphonatocalix[4]arene (**1**) and 1,3-di-O-phosphonatocalix[4]arene (**2**) were synthesised as per the literature methods, in particular for **1** the method for use *in vivo* was used [23].

Compound **1** is well known for its capacity to complex basic amino-acids both in solution [24], for lysine K_ass_ = 1356 M^−1^ and for arginine K_ass_ = 1546 M^−1^, the solid state structures of both complexes have been determined. That of the lysine complex shows the hydrophobic chain embedded in the macrocyclic cavity, this structure is in complete concordance with that obtained in solution [25]. For the arginine complex, four different orientations for the amino acid side chain are observed within the cavity [26]. In the case of **2** binding of basic amino acids occurs, but is dependent on the presence of co-solute cations [27], the solid-state structure with lysine has been determined and shows complexation at the phosphonato groups externally to the macrocyclic cavity [28]. The relevant physical data on the four serum albumins in the current work are given in Table 1 below.

**Table 1:** Physical molecular data of different albumin species.

	BSA	HSA	PSA	SSA
Molar Mass	69,323.44	66,472.21	69,692.17	69,188.28
Iso Electric Point	5,82	5,67	6,08	5,8
Amino Acid number	607	585	607	607
Lysine Residues	60	59	58	61
Arginine Residues	26	24	29	25
Histidine Residues	17	16	19	18
Aspartic Acid Residues	40	36	36	44
Glutamic Acid Residues	59	62	61	56
Homology	100%	76%	80%	92%
Structure (reference)	[10]	[29]	ND *	ND *
Accession Number PDB	PDB: 4F5S	PDB ID: 1AO6		
Accession Number NCBI	NP_851335.1	PMID: 10388840	NP_001005208.1	NP_001009376.1

* ND: Not Determined.

The serum albumins show molecular masses in the range 65 to 70 kD and have iso-electric points below pH 7. The solid-state structures show the presence of binding pockets, in general lined with basic aminoacids, and indeed the serum albumins are known as transporters of anioinic organic molecules, but also are capable of transporting various metal cations. While there is a good homology between the serum albumins, the differences, particularly in the anion binding pockets, led us to consider that differential binding with the two anionic calixarenes **1** and **2** might occur.

**Figure 1:**
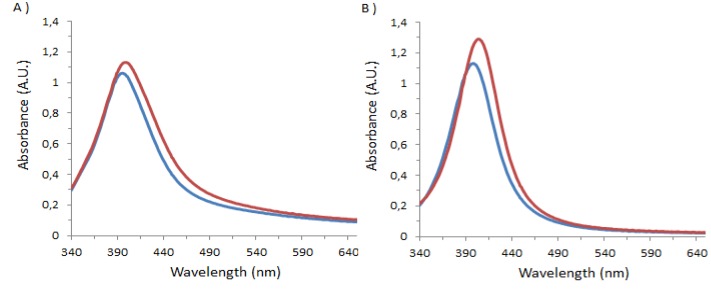
UV-Visible spectra of (**A**) **1**_Ag_NP or (**B**) **2**_Ag_NP mixed with DI water (in blue) or with BSA (in red).

Previous studies, using Electrospray Mass Spectrometry (ES/MS) have shown that **1** binds strongly to the various serum albumins, and that several binding sites are present. The K_ass_ values vary from 7.69 × 10^5^ M^−1^ to 3.85 × 10^5^ M^−1^ to 0.33 × 10^5^ M^−1^ for binding to BSA, and sequential but differentiated binding occurs for the other serum albumins [19]. However there is some doubt as to whether partial denaturation may occur under the particular conditions required for the ES/MS experiment which has led us to attempt to use true solution methods. The capped silver nanoparticles using **1** and **2** were prepared as previously described [20]. Both systems showed a strong plasmon resonance absorption at 390 nm and 398 nm, respectively. Initial experiments confirmed by visible spectroscopy the complexation of BSA on the surface of the capped nanoparticles, this was accompanied by a shift in the resonance band to higher frequency, for **1** to 398 nm and for **2** to 404 nm and a clear increase in the intensity of the resonance band, Figure 1, below. In neither case was a band typical of aggregation observed.

**Figure 2:**
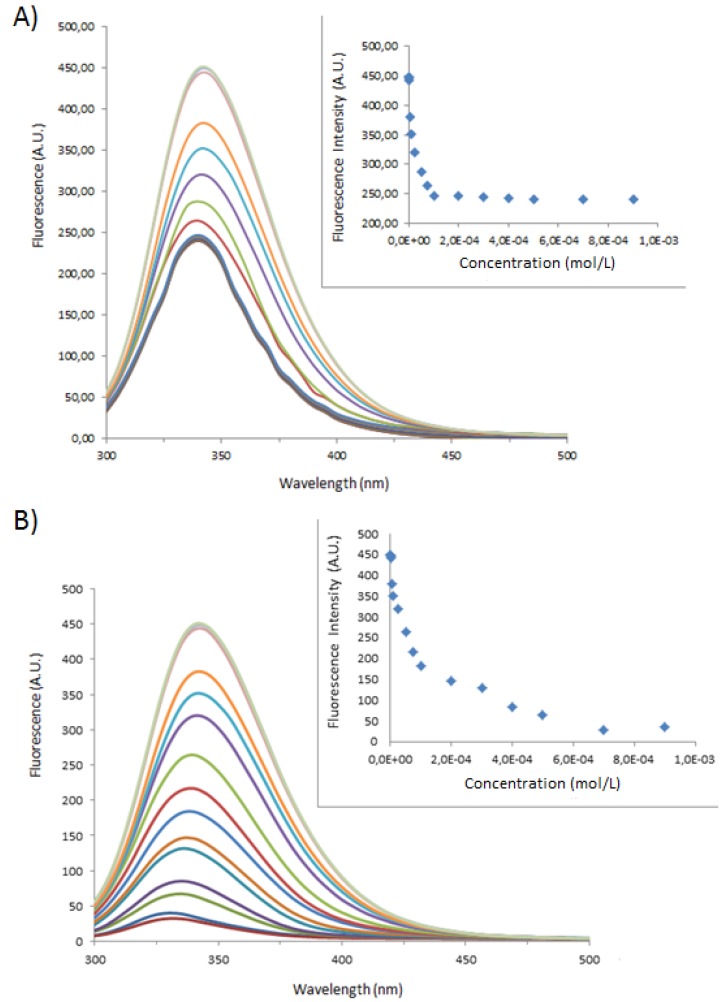
Fluorescence spectra of BSA at 1 × 10^−5^ M with varying concentrations of (**A**) **1** or (**B**) **1**_Ag_NP. In the inset, BSA maximum fluorescence intensity as a function of (**A**) **1** or (**B**) **1**_Ag_NP. (— DI Water; **—** 1 × 10^−6^ M; **—** 2.5 × 10^−6^ M; **—** 5 × 10^−6^ M; **—** 1 × 10^−5^ M; **—** 2.5 × 10^−4^ M; **—** 5 × 10^−5^ M; **—** 7.5 × 10^−5^ M; **—** 1 × 10^−4^ M; **—** 2 × 10^−4^ M; **—** 3 × 10^−4^ M; **—** 4 × 10^−4^ M; **—** 5 × 10^−4^ M; **—** 7 × 10^−4^ M; **—** 9 × 10^−4^ M).

**Figure 3:**
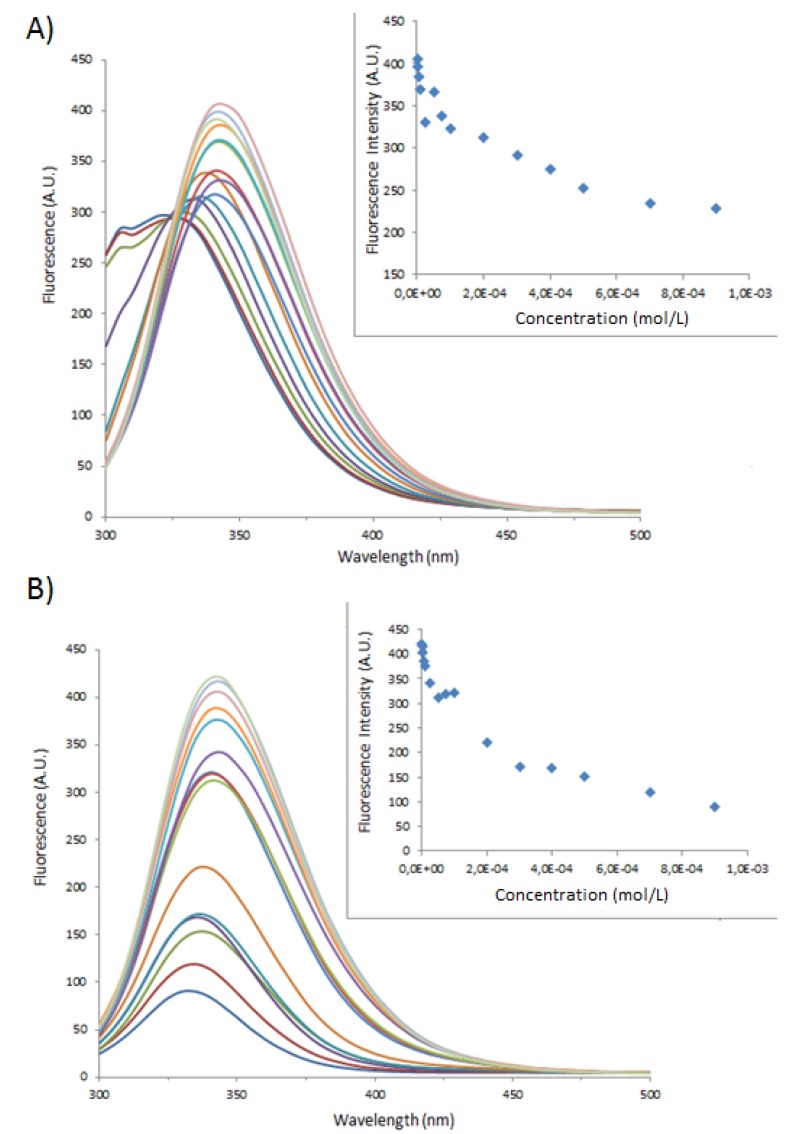
Fluorescence spectra of BSA at 1 × 10^−5^ M varying concentrations of (**A**) **2** or (**B**) **2**_Ag_NP. In the inset, BSA maximum fluorescence intensity as a function of (**A**) **2** or (**B**) **2**_Ag_NP. (— DI Water; **—** 1 × 10^−6^ M; **—** 2.5 × 10^−6^ M; **—** 5 × 10^−6^ M; **—** 1 × 10^−5^ M; **—** 2.5 × 10^−4^ M; **—** 5 × 10^−5^ M; **—** 7.5 × 10^−5^ M; **—** 1 × 10^−4^ M; **—** 2 × 10^−4^ M; **—** 3 × 10^−4^ M; **—** 4 × 10^−4^ M; **—** 5 × 10^−4^ M; **—** 7 × 10^−4^ M; **—** 9 × 10^−4^ M).

The use of fluorescence spectroscopy,Figure 2 and Figure 3 below, allowed the determination of the association constants, by the well-known method of Benesi-Hildebrand [30]. Starting from the equation:

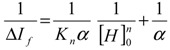
(1)
in which, ΔIf = If_h−g_– If_g_– If_h_, where, If_h−g_, If_g_ and If_h_ are the fluorescence intensity of the host–guest complex, the guest molecule and the host molecule, respectively. [H]_0_ is the original concentration of the calix[n]arenes, K is the inclusion constant, n is the number of host molecule (s) in a complex and α is a constant. Then, it is possible to determine the inclusion constant by plotting 1/ΔIf *versus* 1/[H]_0_^n^ for different values n, the value of n that results a straight line can be taken as the number of host molecules.

The fluorescence emission of the tryptophan amino acids is quenched with increasing concentrations of **1**, **2**, **1**_Ag_NP and **2**_Ag_NP, in the insets the maximum fluorescence is plotted as a function of the concentration of the various systems. While the spectra and maximum emission values for **1** and **1**_Ag_NP, follow typical binding curves; those for **2** show red shifts at high concentrations of **2**, suggesting an increase in the polarity of the local environment of the tryptophan residues.

The observed value for **1** at 7.2 × 10^5^ is reasonably close to the average value obtained from ES/MS experiments, at 3.95 × 10^5^, however for **2** the k_ass_ value is much lower at 1.0 × 10^5^ [19], this difference undoubtedly reflects the capacity of **1** to bind the basic amino acid side chains within the macrocyclic cavity [26], whereas **2** binds the basic amino acids externally to the cavity [28].

The calculated association constant values for the capped nanoparticles are considerably lower (2.4 × 10^4^ M^−1^ for **1**_Ag_NP), in particular in the case of **2**_Ag_NP (3.5 × 10^3^ M^−1^ for **2**_Ag_NP) implying that at least one of the stronger binding pockets on BSA is not available for interaction with the anionic calix[4]arene-capped nanoparticles. The observed results and stoichiometries are summarized in Table 2, below. It is interesting to note that the rule of thumb calculation that the concentration at half-height is equal to the K_dis_ value gives values which are at a first approximation close to those calculated from the method of Benesi [30].

**Table 2:** Stoichiometry S and Association Constants K_ass_ of different samples toward BSA. K_ass_ has been calculated according 2 different methods, that of Benesi, [30] and a simplistic method where K_dis_ is calculated as the half height of the binding curve and K_ass_ is taken simply as the inverse.

Sample	Type of Bovine Albumin	Stoichiometry determined according to the method of Benesi [30]	K_ass_ determined according to the method of Benesi [30] (M^−1^)	K_ass_ apparent (M^−1^)
**1**	BSA	1 : 1	71,666	100,000
**1**_Ag_NP	BSA	1 : 1	24,000	16,600
**1**_Ag_NP	BSA-FITC	1 : 1	5,200	4,540
**2**	BSA	1 : 1	10,000	5,500
**2**_Ag_NP	BSA	1 : 1	3,500	5,500
**2**_Ag_NP	BSA-FITC	1 : 1	5,400	3,300

**Figure 4:**
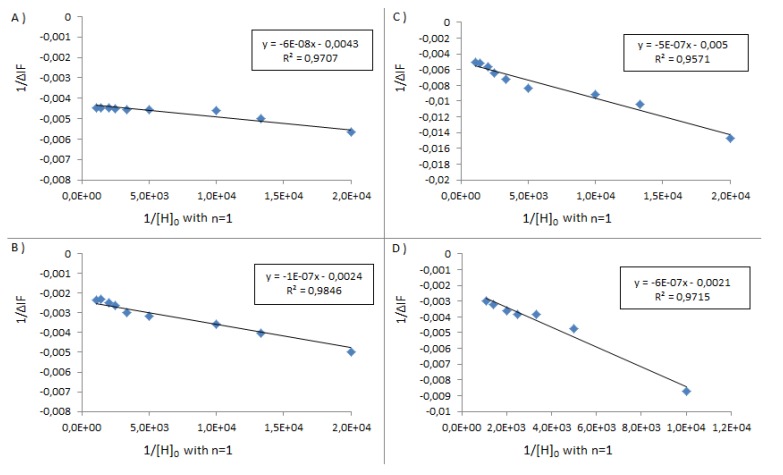
Graph of 1/ΔIf *versus* 1/[H]_0_ for BSA above (**A**) **1**, (**B**) **1**_Ag_NP, (**C**) **2**, (**D**) **2**_Ag_NP. In the inset, the straight line equation with the coefficient of determination R^2^.

In Figure 4 below are given the plots of 1/ΔIf *versus* 1/[H]_0_ as per Benesi, [30] this allows the calculation of an approximate stoichiometry of the complexation events, the best straight lines are obtained for 1:1 stoichiometries. It must be underlined that such deductions should be treated with some care as the exact nature of the binding events between serum albumins and nanoparticles are not completely clear.

**Figure 5:**
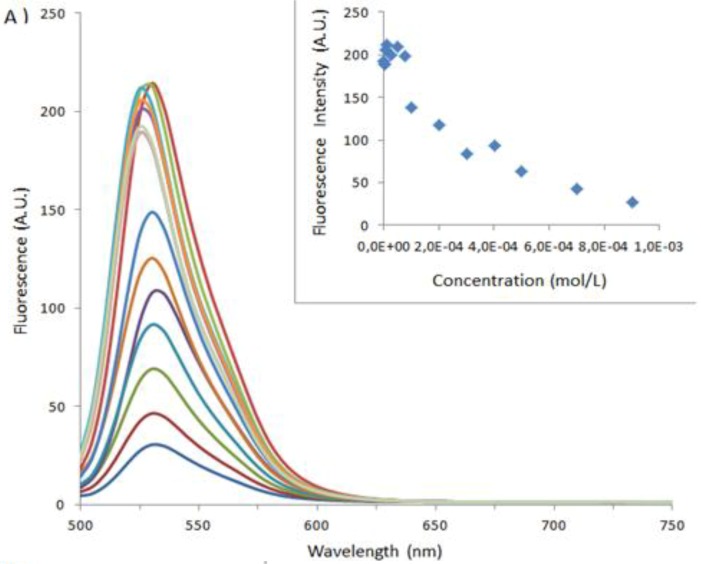

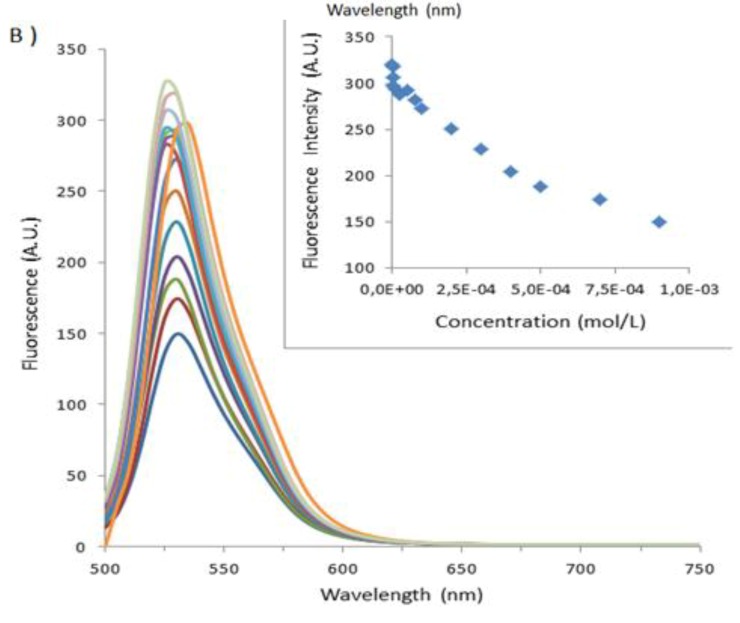
Fluorescence spectra of BSA at 1 × 10^−5^ M varying concentrations of (**A**) **1**_Ag_NP or (**B**) **2**_Ag_NP. In the inset, BSA maximum fluorescence intensity as a function of (**A**) **1** _Ag_NP or (**B**) **2**_Ag_NP. (— DI Water; **—** 1 × 10^−6^ M; **—**2.5 × 10^−6^ M; **—**5 × 10^−6^ M; **—** 1 × 10^−5^ M; **—** 2.5 × 10^−4^ M; **—** 5 × 10^−5^ M; **—** 7.5 × 10^−5^ M; **—** 1 × 10^−4^ M; **—** 2 × 10^−4^ M; **—** 3 × 10^−4^ M; **—** 4 × 10^−4^ M; **—** 5 × 10^−4^ M; **—** 7 × 10^−4^ M; **—** 9 × 10^−4^ M).

**Figure 6:**
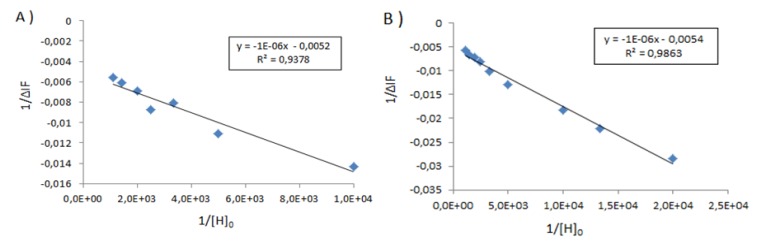
Graph of 1/ΔIf *versus* 1/[H]_0_ for BSA-FITC above (**A**) **1**_Ag_NP, (**B**) **2**_Ag_NP. In the inset, the straight line equation with the coefficient of determination R^2^.

We were hopeful that the use of FITC capped albumins would open up a second spectroscopic probe with higher inherent fluorescence intensity than that of the aromatic amino acids. As there are only two fluorescent tryptophan, 21 fluorescent tyrosine and 30 fluorescent phenylalanine amino acids; this should allow enhancement of the fluorescence signal and thus diminution of the minimum detectable limit. The results are shown in Figure 5 below, and the global results are summarized in Table 2. Here the binding is far lower than that observed with BSA alone, at 5.2 × 10^3^ M^−1^ for **1**_Ag_NP and 5.4 × 10^3^ M^−1^ for **2**_Ag_NP, Figure 6. On reflection this is not surprising as FITC selectively couples to lysine residues which are one of the main recognition sites for the anionic calix[4]arenes [11]. 

Initial experiments with the complexation of the anioinic calix[4]arene capped silver nanoparticles with different serum albumins showed a time dependent variation in the intensities of the plasmon resonance peak. Subsequent experiments showed that this variation was temperature dependent and quite rapid, taking place over about 20 minutes. The known values of the denaturing temperatures of the serum albumins, *i.e.*, bovine serum albumin (BSA), human serum albumin (HAS), porcine serum albumin (PSA) and sheep serum albumin (ssa) are given below in Table 3 [31,32,33,34]. The quite large span of temperatures for BSA is known to be associated with partial unfolding of the protein [31].

**Table 3:** Denaturation temperatures of different albumins.

	BSA	HSA	PSA	SSA
Denaturation Temperature (°C)	50–80	50	68	60

**Figure 7:**
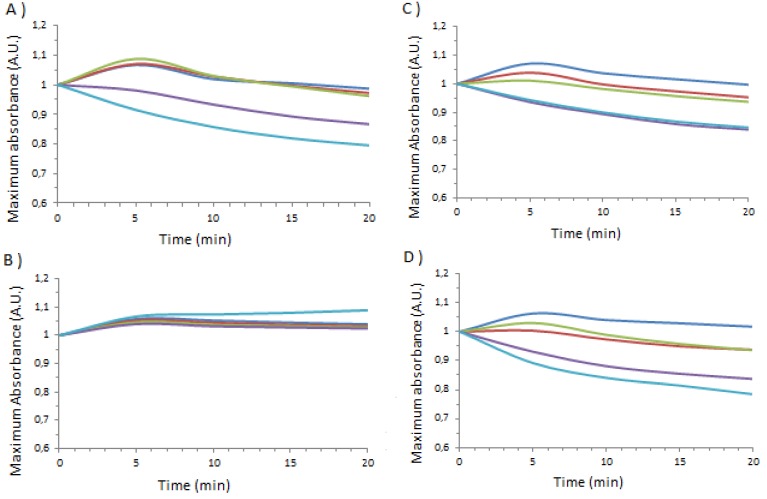

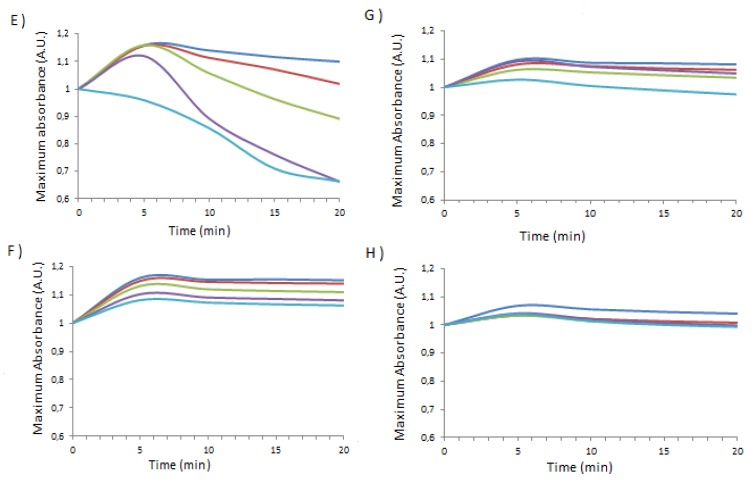
Maximum absorbance, normalized to 1 at time zero of calix[n]arene capped silver nanoparticles mixed with different albumin species as a function of time and temperature (thick blue: 20 °C, red: 30 °C, green: 40 °C, purple: 50 °C and sky blue: 60 °C). On the left, **1**_Ag_NP mixed with BSA (**A**), HSA (**B**), PSA (**C**), and SSA (**D**); on the right **2**_Ag_NP mixed with BSA (**E**), HSA (**F**), PSA (**G**) and SSA (**H**).

In Figure 7, above are shown the temporal variations in the intensity of the plasmon resonance peak as a function of temperature for the two nanoparticles in the presence of different serum albumins. The relevant percentage changes after 5 min and 15 min are summarized in Table 4 and Table 5, respectively. It can clearly be seen that it is possible to differentiate between species using this data, for example at 50 °C for BSA with **1**_Ag_NP there is a change of −8% after 5 min −11% after 15 min, for **2**_Ag_NP the changes are -3% after 5 min and −32% after 15 min whereas for PSA the same data gives 13%, −0.5% for **1**_Ag_NP and −15% and −0.5% for **2**_Ag_NP. The diminuition in the intensity of the plasmon resonance absorption of **2**_Ag_NP in the presence of BSA is quite singular at over 30% for temperatures above the initial partial unfolding [31]. It thus becomes possible with a quite simple methodology to discriminate between the species

**Table 4:** Percentage changes of the maximum absorption of calixarenes-capped silver nanoparticles against Serum Albumin a as function of temperature, after 5 min.

Temperature applied (°C)	1_Ag_NP / BSA	1_Ag_NP / HSA	1_Ag_NP / PSA	1_Ag_NP / SSA	2_Ag_NP / BSA	2_Ag_NP / HSA	2_Ag_NP / PSA	2_Ag_NP / SSA
20	0	0	0	0	0	0	0	0
30	0.3	−0.5	−3	−5.62	−0.17	−0.95	−1.55	−3.18
40	1.93	−1.1	−5.6	−3.13	0	−2.5	−3.28	−3.28
50	−8.08	−1.71	−12.61	−12.46	−3.36	−5	−0.55	−2.53
60	−14.26	0.8	−11.95	−16.16	−17.26	−6.81	−6.47	−2.81

**Table 5:** Percentage changes of the maximum absorption of calixarenes-capped silver nanoparticles as function of temperature, after 15 min.

Temperature applied (°C)	1_Ag_NP / BSA	1_Ag_NP / HSA	1_Ag_NP / PSA	1_Ag_NP / SSA	2_Ag_NP / BSA	2_Ag_NP / HSA	2_Ag_NP / PSA	2_Ag_NP / SSA
20	0	0	0	0	0	0	0	0
30	−0.78	−0.7	−4.14	−7.74	−4.03	−1.13	−1.55	−3.15
40	−1.04	−1.21	−5.81	−6.93	−13.71	−3.55	−3.28	−3.92
50	−11.17	−1.64	−15.46	−16.95	−31.81	−5.98	−0.55	−3.73
60	−18.52	3.3	−14.57	−20.87	−36.38	−7.63	−6.47	−4.4

## 3. Experimental

### 3.1. Synthesis

*para*-Sulphonatocalix[4]arene (**1**) was synthesized as per the literature method and physical characteristics correspond to the literature values [23]. The diphosphonate derivative 2 was synthesized as per the method of Markovsky and Kalchenko [35], and all spectral values were in accord with their reported values.

### 3.2. Nanoparticle Preparation and Characterization

The procedure of Xiong [20] was slightly modified as follows: 1 × 10^−2^ M AgNO_3_ solution (10 mL) was added to deionized water (80 mL). To this solution, 1 × 10^−2^ M calix[n]arene aqueous solution (10 mL) was added as stabilizer and the mixture stirred during 30 min. Then, NaBH_4_ (44 mg) was added to the solution. The calix[n]arene capped silver colloidal suspensions were characterized by UV-Visible absorption assays. The change in absorbance between 340 nm and 650 nm was monitored, using a 96 titer well visible spectrometer, (BioTek Power Wave 340, Bad Friedrichshall, Germany).

### 3.3. Fluorimetry Experiments

All the fluorescence spectra were measured at 20 °C. The albumin was diluted at a final concentration of 1 × 10^−5^ M and was mixed with a varying concentration of calix[n]arene (or calix[n]arene-capped silver nanoparticles) from 9 × 10^–4^ to 1 × 10^−6^ M. Fluorescence spectra and fluorescence intensities were measured on a model F-6500 fluorescence spectrophotometer (Hitachi, Tokyo, Japan) using a 1(W) × 3(D) × 35(H) mm micro-quartz cell. The slits for the emission monochromators were fixed at 5.0 nm. The excitation wavelength was set at 290 nm for bovine serum albumin (BSA) and 495 nm for the BSA-fluorescein conjugate. The emission spectra were monitored from 300 to 500 nm for BSA and 500 to 750 nm for the BSA-fluorescein conjugate.

### 3.4. Thermal Complexation Titrations

All albumins were purchased from Sigma Aldrich (Sigma Aldrich, Saint-Quentin, France), and used without further purification The calix[n]arene capped silver colloidal suspensions and the albumin were heated independently 10 min before mixing. Then, the complexation between albumin and calix[n]arene capped silver colloidal suspensions was monitored using a thermoregulated cell accessory for the Jasco spectrophotometer FP-6200 (Tokyo, Japan). The value at 490 nm corresponding to the absorption band of aggregated nanoparticles was measured, every 5 minutes during 20 min at 20, 30, 40, 50 and 60 °C.

## 4. Conclusion

We have studied the use of fluorescence spectroscopy and visible spectroscopy to obtain discrimination between the behaviour of four different types of serum albumins. The temperature and time dependent variation in the intensity of the plasmon resonance peak in calix[n]arene capped nanoparticles provides a suitable method to differentiate between different animal species.
